# Supplementary material for: Resolving the full spectrum of human genome variation using Linked-Reads
Source: Genome Res. 2019 Apr;29(4):635–45. doi: 10.1101/gr.234443.118 (PMC6442396; doi:10.1101/gr.234443.118)
Supplement: Supplemental Material [file supp_gr.234443.118_Supplemental_longranger-2.2.zip › longranger-2.2.tar.bz2!/lr2.2-public/lib/stan/cmdstan/stan/doc/api/html/agrad_2rev_2matrix_2sort_8hpp_source.html]

Stan: src/stan/agrad/rev/matrix/sort.hpp Source File


|  |  |
| --- | --- |
|  | Stan  2.5.0  probability, sampling & optimization |


- Main Page
- Related Pages
- Namespaces
- Classes
- Files

- File List
- File Members

All Classes Namespaces Files Functions Variables Typedefs Enumerations Enumerator Friends Macros Pages

- src
- stan
- agrad
- rev
- matrix

sort.hpp

Go to the documentation of this file.

1 #ifndef STAN\_\_AGRAD\_\_REV\_\_SORT\_HPP

2 #define STAN\_\_AGRAD\_\_REV\_\_SORT\_HPP

3

4 #include <valarray>

5 #include <stan/agrad/rev/var.hpp>

6 #include <stan/agrad/rev/internal/v\_vari.hpp>

7 #include <stan/agrad/rev/operators/operator\_greater\_than.hpp>

8 #include <stan/agrad/rev/operators/operator\_less\_than.hpp>

9 #include <vector>

10 #include <stan/math/matrix/Eigen.hpp>

11 #include <algorithm> // std::sort

12 #include <functional> // std::greater

13

14 namespace stan {

15  namespace agrad {

16

24  inline std::vector<var> sort\_asc(std::vector<var> xs) {

25  std::sort(xs.begin(), xs.end());

26  return xs;

27  }

28

36  inline std::vector<var> sort\_desc(std::vector<var> xs) {

37  std::sort(xs.begin(), xs.end(), std::greater<var>());

38  return xs;

39  }

40

48  template <int R, int C>

49  inline typename Eigen::Matrix<var,R,C> sort\_asc(Eigen::Matrix<var,R,C> xs) {

50  std::sort(xs.data(), xs.data()+xs.size());

51  return xs;

52  }

53

61  template <int R, int C>

62  inline typename Eigen::Matrix<var,R,C> sort\_desc(Eigen::Matrix<var,R,C> xs) {

63  std::sort(xs.data(), xs.data()+xs.size(), std::greater<var>());

64  return xs;

65  }

66

67  }

68 }

69 #endif

operator\_greater\_than.hpp

stan::agrad::sort\_asc

std::vector< fvar< T > > sort\_asc(std::vector< fvar< T > > xs)

**Definition:** sort.hpp:18

v\_vari.hpp

stan::agrad::sort\_desc

std::vector< fvar< T > > sort\_desc(std::vector< fvar< T > > xs)

**Definition:** sort.hpp:26

var.hpp

Eigen.hpp

operator\_less\_than.hpp


---

[ Stan Home Page ]

*© 2011–2014,
Stan Development Team.*
